# Moyamoya-like cerebrovascular disease in a child with a novel mutation in myosin heavy chain 11

**DOI:** 10.1212/WNL.0000000000004828

**Published:** 2018-01-16

**Authors:** Annette Keylock, Ying Hong, Dawn Saunders, Ebun Omoyinmi, Ciara Mulhern, Derek Roebuck, Paul Brogan, Vijeya Ganesan, Despina Eleftheriou

**Affiliations:** From UCL GOS Institute of Child Health (A.K., Y.H., E.O., C.M., P.B., V.G., D.E.); and Great Ormond Street Hospital (D.S., D.R.), London, UK.

Heterozygous mutations in the *MYH11* gene affecting the C-terminal coiled-coil region of the smooth muscle myosin heavy chain, a contractile protein of smooth muscle cells (SMC), have been described to cause thoracic aortic aneurysm or aortic dissection (TAAD) and patent ductus arteriosus (PDA).^1^ Herein we expand the phenotype associated with *MYH11* mutations to include moyamoya-like cerebrovascular disease.

## Case report

**Figure 1:**
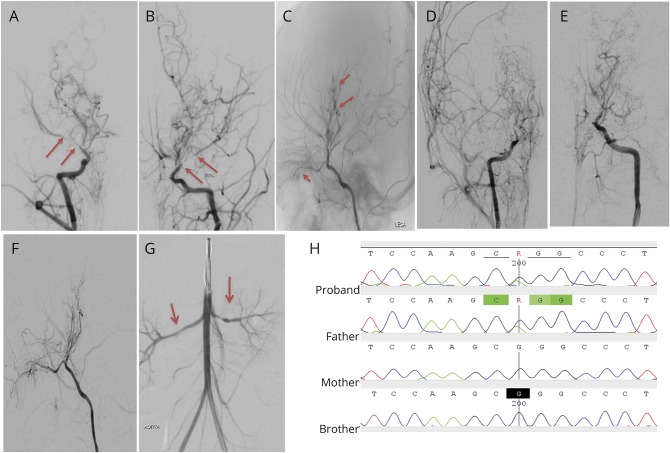
Cerebral arteriopathy associated with a novel heterozygous mutation in *MYH11* (A) Right and (B) left anteroposterior projections of the internal carotid artery (ICA) injections of the cerebral angiogram at presentation demonstrate narrowing of the terminal internal carotid artery (TICA) and straight and long segment narrowing of the M1 and A1 segments of the middle cerebral artery (MCA) (between arrows) and anterior cerebral artery (ACA) bilaterally. Moyamoya collaterals are present on the initial angiogram. (C) Lateral projection of the left ICA injection demonstrates the presence of basal and moyamoya collaterals (short arrows). Four years later, progression of the arteriopathy is seen with (D) further narrowing of the right M1 segment of the MCA and (E, F) occlusion of the left TICA with absent filling of the terminal ACA and MCA arteries. (F) The number of basal and moyamoya collaterals has increased. The pial collaterals in (C) are a result of the pial synangiosis. (G) Bilateral narrowing of the renal arteries seen on digital subtraction renal angiography. (H) Sanger sequencing chromatogram of *MYH11* gene aligned to reference sequence exon 33 of *MYH11* (NM_002474). Line indicates a heterozygous nonsynonymous substitution present in the proband and father c.4604G>A (p.R1535Q) but not in the mother or brother.

A 2-year-old girl of Moroccan nonconsanguineous descent presented with a right-sided hemiparesis and aphasia (figure e-1, links.lww.com/WNL/A36). Brain MRI revealed an acute left anterior and middle cerebral artery territory (ACA/MCA) infarct and prior infarction in the right ACA/MCA territory (figure e-1). The clinical correlate of the latter was an episode of reluctance to use the left hand. Catheter angiography showed bilateral stenosis of the terminal internal carotid artery (TICA)/middle cerebral artery with a collateralization pattern consistent with moyamoya arteriopathy (figure 1, A–F). Visceral digital subtraction angiography revealed narrowing of the mid-aorta and bilateral renal artery stenosis (figure 1G). Echocardiography showed a small PDA. In view of 2 cutaneous café-au-lait spots, genetic screening for neurofibromatosis type 1 was undertaken and was negative; array comparative genomic hybridization was normal. She had no mydriasis, gastrointestinal, bladder, or bowel dysfunction. She was considered to have a generalized vasculopathy with prominent cerebral involvement and she underwent bilateral pial synangiosis. There was radiologic arteriopathy progression over 4 years, with new right frontal infarction, increased stenosis of the right internal carotid artery/MCA, and occlusion of the left TICA with the development of more basal collaterals (figure 1, D–F, and figure e-1). She currently has residual asymmetric tetraparesis with pseudobulbar features and is normotensive with normal cardiac function.

Whole exome sequencing (Methods appendix e-1, links.lww.com/WNL/A38) revealed a novel heterozygous missense mutation in *MYH11* gene NM_002474:c.4604G>A (p.R1535Q) confirmed with Sanger sequencing (figure 1H) present in both the proband and her father and predicted damaging based on SIFT, MutationTaster, and PolyPhen-2 programs. This finding also had implications for the proband's father, for whom annual cardiovascular monitoring was initiated; baseline cardiac MRI and magnetic resonance angiography were normal for him.

To date, a number of vascular disorders have been associated with mutations directly affecting SMC contractile proteins or proteins that disrupt SMC contractility (table e-1, links.lww.com/WNL/A37).^2^ The majority of these conditions are characterized by prominent thoracic aorta involvement.^1–3^ Recently, however, specific mutations, for instance heterozygous missense mutations in *ACTA2* disrupting Arg179, have been shown to associate mainly with cerebrovascular disease.^4^ We now also expand the phenotype associated with heterozygous mutations in *MYH11* to include an occlusive cerebral arteriopathy. We would suggest that screening for cerebrovascular involvement should be recommended for all patients with *MYH11* mutations. This is also clinically relevant as β-blockade may be considered in patients with TAAD and β-blockade may adversely affect systemic blood pressure and therefore perfusion of the brain.

The radiologic phenotype of the observed arteriopathy appears to be different from that associated with *ACTA2.* The *ACTA2* arteriopathy is characterized by an abnormally straight morphology of proximal branches of the circle of Willis, occlusive features, and a paucity of basal collaterals, distinct from classical “moyamoya.”^4,5^ The initial arterial morphology in the *MYH11* patient also shows this straight configuration of the arterial circulation although, in contrast to *ACTA2* patients, the index cases go on to develop profuse basal “moyamoya” collaterals over time. While in *ACTA2* patients the distinctive radiologic signature is readily apparent,^4,5^ it may be that other SMC-related arteriopathies have morphologic signatures that are yet to be recognized.

The missense alteration, R1535Q, in exon 33 of *MYH11* that we identified is likely to affect the communication between the motor domain and the coiled-coil tail of the SM-MHC-11 protein. Similar changes in the SM-MHC-11 protein structure in TAAD lead to reduced myosin motor elasticity, aberrant interactions with actin filaments, SMC shortening, and contractile force generation followed by upregulation of tumor growth factor–β activity.^6,7^ A similar mechanism may be implicated in the pathogenesis of the cerebral arteriopathy we observed with various external triggers contributing to disease development. The lack of a vascular phenotype in the father to date is in line with previous reports that suggested variability of disease onset and progression in relation to TAAD associated with *MYH11*.^6,7^

We emphasize the systemic nature of the vasculopathy associated with *MYH11* mutations and the need for broader than previously suggested vascular surveillance to include the cerebrovascular circulation. The contribution of *MYH11* mutations to isolated cerebral arteriopathy remains to be established. We would propose that *MYH11* testing could be considered in children with moyamoya who have atypical features on cerebral angiography or poor response to pial synangiosis.
